# Nociceptive mechanisms driving pain in a post-traumatic osteoarthritis mouse model

**DOI:** 10.1038/s41598-020-72227-9

**Published:** 2020-09-17

**Authors:** C. J. Alves, M. Couto, D. M. Sousa, A. Magalhães, E. Neto, L. Leitão, F. Conceição, A. C. Monteiro, M. Ribeiro-da-Silva, M. Lamghari

**Affiliations:** 1 Neuro-Skeletal Circuits Group, Instituto de Investigação e Inovação em Saúde (i3S), Universidade do Porto, Rua Alfredo Allen, 208, 4200-135 Porto, Portugal; 2 Instituto de Engenharia Biomédica (INEB), Universidade do Porto, Porto, Portugal; 3 Instituto de Biologia Molecular e Celular (IBMC), Universidade do Porto, Porto, Portugal; 4 Instituto Ciências Biomédicas Abel Salazar (ICBAS), Universidade de Porto, Porto, Portugal; 5 Faculdade de Medicina, Universidade do Porto (FMUP), Porto, Portugal; 6 Serviço de Ortopedia e Traumatologia, Centro Hospitalar São João, Porto, Portugal

**Keywords:** Peripheral nervous system, Osteoarthritis

## Abstract

In osteoarthritis (OA), pain is the dominant clinical symptom, yet the therapeutic approaches remain inadequate. The knowledge of the nociceptive mechanisms in OA, which will allow to develop effective therapies for OA pain, is of utmost need. In this study, we investigated the nociceptive mechanisms involved in post-traumatic OA pain, using the destabilization of the medial meniscus (DMM) mouse model. Our results revealed the development of peripheral pain sensitization, reflected by augmented mechanical allodynia. Along with the development of pain behaviour, we observed an increase in the expression of calcitonin gene-related peptide (CGRP) in both the sensory nerve fibers of the periosteum and the dorsal root ganglia. Interestingly, we also observed that other nociceptive mechanisms commonly described in non-traumatic OA phenotypes, such as infiltration of the synovium by immune cells, neuropathic mechanisms and also central sensitization were not present. Overall, our results suggest that CGRP in the sensory nervous system is underlying the peripheral sensitization observed after traumatic knee injury in the DMM model, highlighting the CGRP as a putative therapeutic target to treat pain in post-traumatic OA. Moreover, our findings suggest that the nociceptive mechanisms involved in driving pain in post-traumatic OA are considerably different from those in non-traumatic OA.

## Introduction

Osteoarthritis (OA), a disease characterized by articular cartilage degeneration, subchondral bone changes, osteophyte formation, and synovitis, represents one of the major health burdens in the industrialized world^1^. Despite more frequent in the elderly population, OA can also affect, with even more devastating effects, younger individuals as a consequence of genetic risk factors, obesity, occupational or recreational activities, and joint skeletal injuries^2^. Therefore, the management of young adult patients with OA presents a great challenge. The knee trauma, particularly related to chondral and instability injuries, is the major cause of OA in young adult patients^2^. Meniscal tears and ligament damage are well-established risk factors for articular cartilage degeneration, and it is estimated that around 50% of patients diagnosed with any of these conditions will in the long-term have pain and functional impairment caused by knee OA^3^.

In OA, pain is the main cause of reduced patients’ wellbeing and the major reason to seek medical care. The available therapies to treat OA pain (NSAIDs, opiates, corticosteroids) are largely inadequate, due to their limited efficacy, particularly for severe pain, and the plethora of safety issues that arise from prolonged treatment. In the situation of uncontrolled pain, total joint replacement is the ultimate alternative^4^. This drastic solution, although effective in older patients, should be considered with caution in younger and active patients. In fact, the restriction to high-impact activities following arthroplasties, as well as, issues related to the implant survival and the complexity of future revision surgery are major drawbacks of arthroplasties in young patients^2^. Thus, there is an urgent need to understand OA pain mechanisms, to support the development of novel and effective therapies.

Preclinical animal models have played a key role in the investigation of OA pathophysiology. OA animal models are commonly categorized into (i) spontaneous, which includes the naturally occurring and the genetic models, (ii) chemically-induced, and (iii) surgically-induced^5,6^. The naturally occurring models closely mimic the progression of non-traumatic OA in humans as a result of natural wear and tear throughout life, but have the drawback of imposing long progression time, high-costs, and variability in severity and onset of the disease^6^. The use of genetically modified models allows overcoming the variability in severity and onset of the naturally occurring models, however, possible confounding symptoms may result from the genetic alterations. The chemically-induced OA models are obtained by the injection of inflammatory or toxic compounds into the joint, compromising their structure and function. These are rapidly evolving joint degenerative models that are not suitable to study the slow progressive onset of human OA^6^. The OA animal models induced by the surgically joint impairment, such as the destabilization of the medial meniscus (DMM) model, present OA characteristics that closely mimic the disease progression in humans after traumatic injury^6,7^. In the DMM model, the sectioning of the meniscotibial ligament results in the impairment of the medial meniscus function and consequent joint destabilization^8^. Joint lesions have been reported to occur at 4 weeks after DMM surgery, and OA progress from moderate to severe between 8 and 12 weeks post-surgery^6,7,9^.

OA pain is a complex and dynamic process that involves not only structural and biochemical alterations in several components of the joint, such as cartilage, synovium, and bone, but also entails alterations in pain signalling pathways in the peripheral and central nervous system^10^. The research in OA has mostly focused on the mechanisms involved in the joint destruction. However, the mechanisms that mediate pain in progressive post-traumatic OA remain to be clarified.

Currently, it is not known whether OA diseases with different onset and progression patterns will share similar nociceptive mechanisms. For instance, the collagenase-induced OA model displays both mechanical allodynia (a pain sensation provoked by an usually innocuous stimulus) and thermal hypersensitivity, while the DMM model (post-traumatic model) shows only mechanical allodynia (reviewed by^11^). This evidence suggests the involvement of distinct pain pathways between different OA phenotypes. Understanding the nociceptive mechanisms underlying joint trauma-induced OA will support the development of specific and effective therapies, delaying invasive procedures that compromise the mobility of young adult individuals.

In this study, we addressed putative nociceptive mechanisms involved in trauma-induced OA pain, using the DMM model. We focused on the development of both peripheral and central sensitization, and analysed the involvement of synovitis and neuropathic mechanisms.

## Results

### Pain-related behaviour in the OA post-traumatic DMM model

The development of pain in OA animal models is integral to its validation and utility as models for translational research^12^. Rodent OA models present weight-bearing asymmetry and mechanical allodynia, resembling OA patients. Alterations in innate behaviour and gait pattern have been also used as indicators of pain in these animal models^12^.

**Figure 1:**
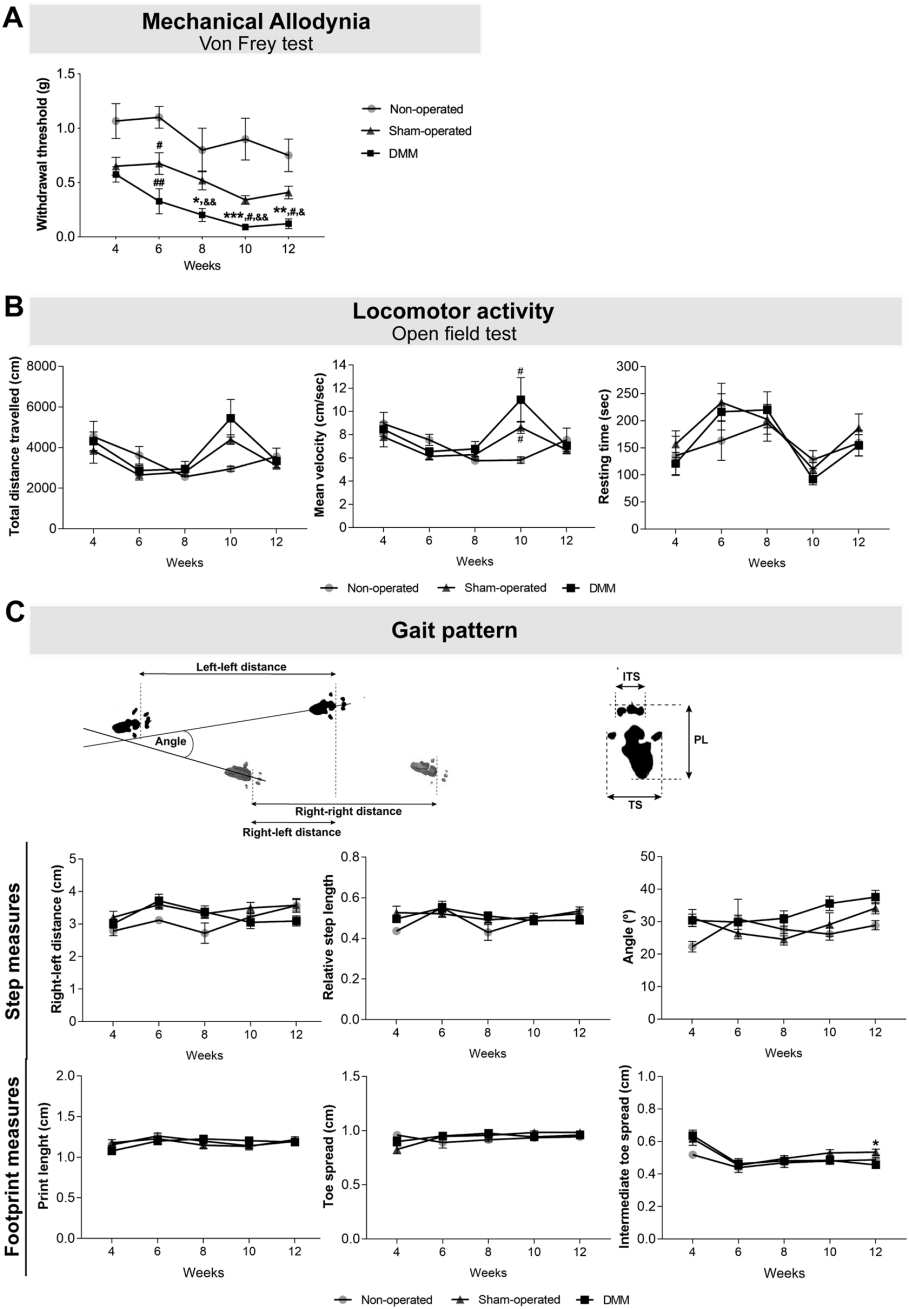
The OA post-traumatic DMM model was characterized by the presence of mechanical allodynia. Mechanical allodynia (**A**), and alterations in locomotor activity (**B**) and gait pattern (**C**) were assessed 4, 6, 8, 10 and 12 weeks after the knee joint traumatic lesion. The Von Frey data analysis showed that DMM mice presented increased mechanical allodynia when compared with sham-operated, starting at week 8. No effects were observed in the locomotor activity. A decrease in the ITS was observed in the DMM mice. Results are presented as mean ± SEM, n = 5 in the control group and n = 8 in the sham-operated and DMM groups. *Indicates differences between sham-operated and DMM groups; ^#^indicates differences when compared with non-operated group; ^&^indicates differences when compared with week 4; *p < 0.05; **p < 0.01; ***p < 0.001; ^#^*p* < 0.05; ^##^*p* < 0.01; ^&^*p* < 0.05; ^&&^*p* < 0.01. *ITS* intermediate toe spread, *PL* print length, *TS* toe spread.

In this study, the Von Frey data analysis revealed that 8 weeks after DMM surgery mice presented increased mechanical allodynia when compared with the non-operated group (p = 0.026), and at week 10 and 12 when compared with non-operated and sham-operated mice (10 weeks: sham-operated p < 0.001 and non-operated p = 0.047; 12 weeks: sham-operated p = 0.004 and non-operated p = 0.042) (Fig. 1A). Moreover, DMM mice presented increased mechanical allodynia at week 8, 10 and 12 post-surgery when compared with week 4 (week 8 p = 0.008; week 10 p = 0.001: week 12 p = 0.015).

Instead, the analysis of the data from the open field test showed no differences, at each analysed time point, between the DMM and the control groups (non-operated and sham-operated mice) concerning (i) distance travelled, (ii) mean velocity and (iii) time spent immobile (Fig. 1B). These results indicate that, even at 12 weeks post-DMM surgery, the locomotor activity was not affected. Regarding gait pattern, no effects were observed in the evaluated measures: (i) right (affected)-left paw distance, (ii) angle between consecutive paw prints and (iii) relative step length step (Fig. 1C). However, the analysis of the footprint revealed that ITS was decreased in the DMM mice when compared with the sham-operated mice (p = 0.019) (Fig. 1C), indicating that mice foot placement was impaired.

### Joint morphological and histological alterations in the OA post-traumatic DMM model

**Figure 2:**
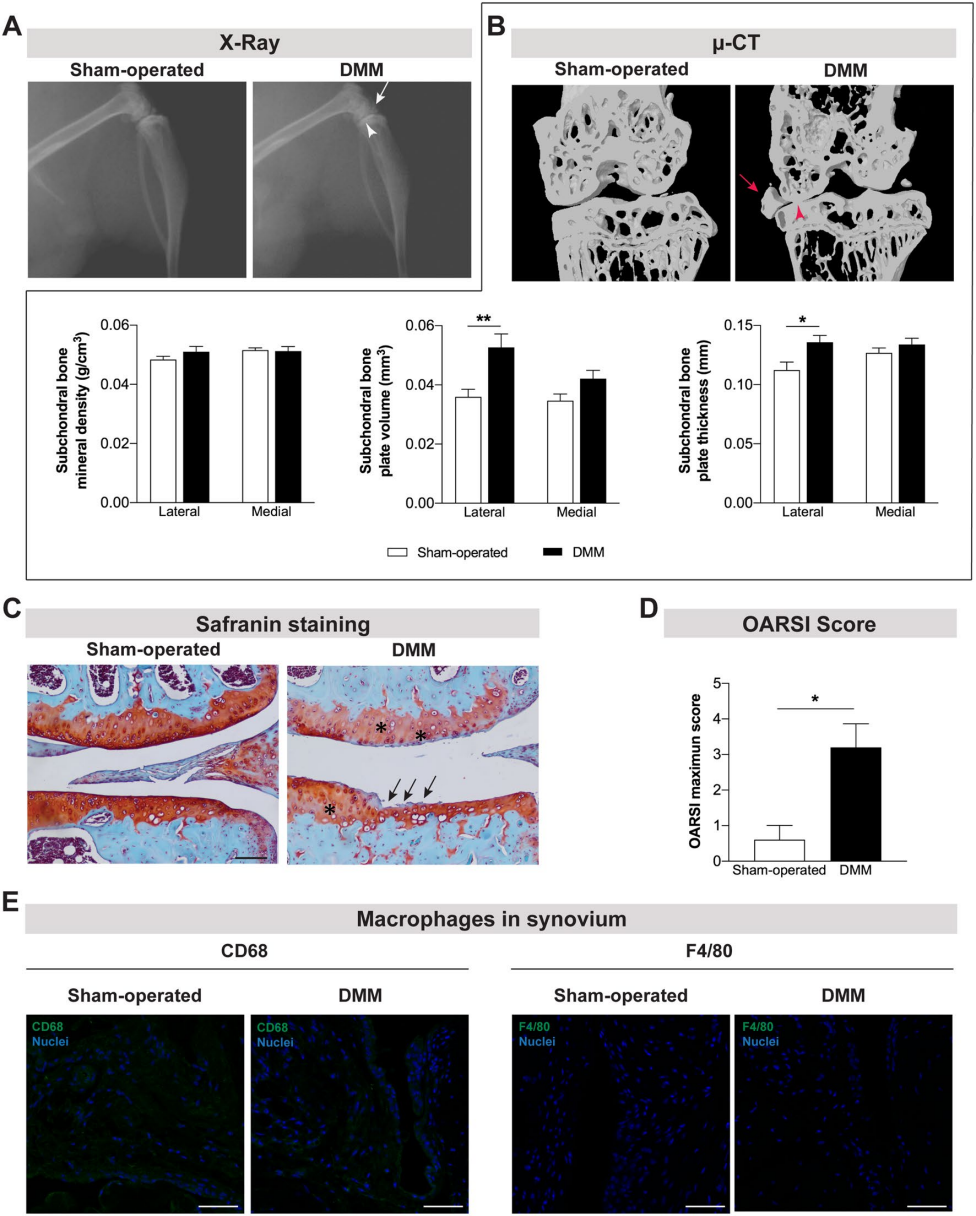
Joint damage was observed 12 weeks after the induction of the OA post-traumatic DMM model. X-ray (**A**) and μ-CT (**B**) images were acquired 12 weeks post-surgery in sham-operated and DMM mice. The narrowing of the knee joint space (arrowhead) and the formation of osteophytes (arrow) were observed in both technical approaches. μ-CT quantitative analyses were also performed (**B**). Increased subchondral bone volume and subchondral bone thickness were observed in the DMM mice. The safranin O staining (**C**) revealed intense fibrillation and thinning of the cartilage (arrows) and lower intensity in red staining (proteoglycan) of cartilage (highlighted with *) in the DMM mice (scale bar = 100 μm). A higher OARSI maximum score of articular cartilage was calculated for the DMM mice (**D**). The infiltration of synovium by macrophages was assessed by the analysis of CD68 and F4/80 expression and no macrophages were observed (**E**) (scale bar = 50 μm). Results are presented as mean ± SEM, n = 5 per group. *p < 0.05, **p < 0.01.

Currently, it is well established that the pathophysiology of OA involves, in addition to cartilage lesion, damage in other joint tissues. Alterations in the subchondral bone and the synovial inflammation have been shown in both patients and animal models^13^. In the present study, the X-ray and μ-CT images acquired 12 weeks post-DMM surgery revealed the narrowing of the knee joint space and the formation of osteophytes (Fig. 2A,B). Further quantitative μ-CT analyses uncovered the development of subchondral bone sclerosis in the tibia of DMM mice, as indicated by the increase in subchondral bone volume (p = 0.0039) and thickness (p = 0.0171) (Fig. 2B). The safranin O staining, a basic stain that binds cartilage proteoglycans, revealed cartilage damage ranging from intense fibrillation to thinning of the articular cartilage in the DMM mice (Fig. 1C). Moreover, the knee sections of DMM mice showed a lower intensity in the red staining of cartilage when compared with sham-operated mice, suggesting the loss of proteoglycan content (Fig. 1C). These alterations in the articular cartilage were traduced in significantly higher OARSI score in the DMM when compared with sham-operated mice (p = 0.038) (Fig. 1D). Overall, the degradation of cartilage, the narrowing of the joint space, and the development of osteophytes and subchondral bone sclerosis support the induction of an OA phenotype at 12 weeks post-DMM surgery.

As above mentioned, increasing evidence suggests that synovial membrane inflammation (characterized by the infiltration of mononuclear cells, thickening of the synovial lining layer and production of inflammatory cytokines) is an important mediator in the pathogenesis of both OA structural degeneration and OA pain^14^. In this experiment, we analysed the synovium for the infiltration by immune cells previously described in synovitis^14^. Our results revealed no infiltration by immune cells, such as macrophages (Fig. 1E), T cells and B cells (data not shown) at 12 weeks after the knee joint traumatic injury. Positive-staining control images are provided as supplementary data (Fig. S1).

### Alteration in the joint innervation in the OA post-traumatic DMM model

**Figure 3:**
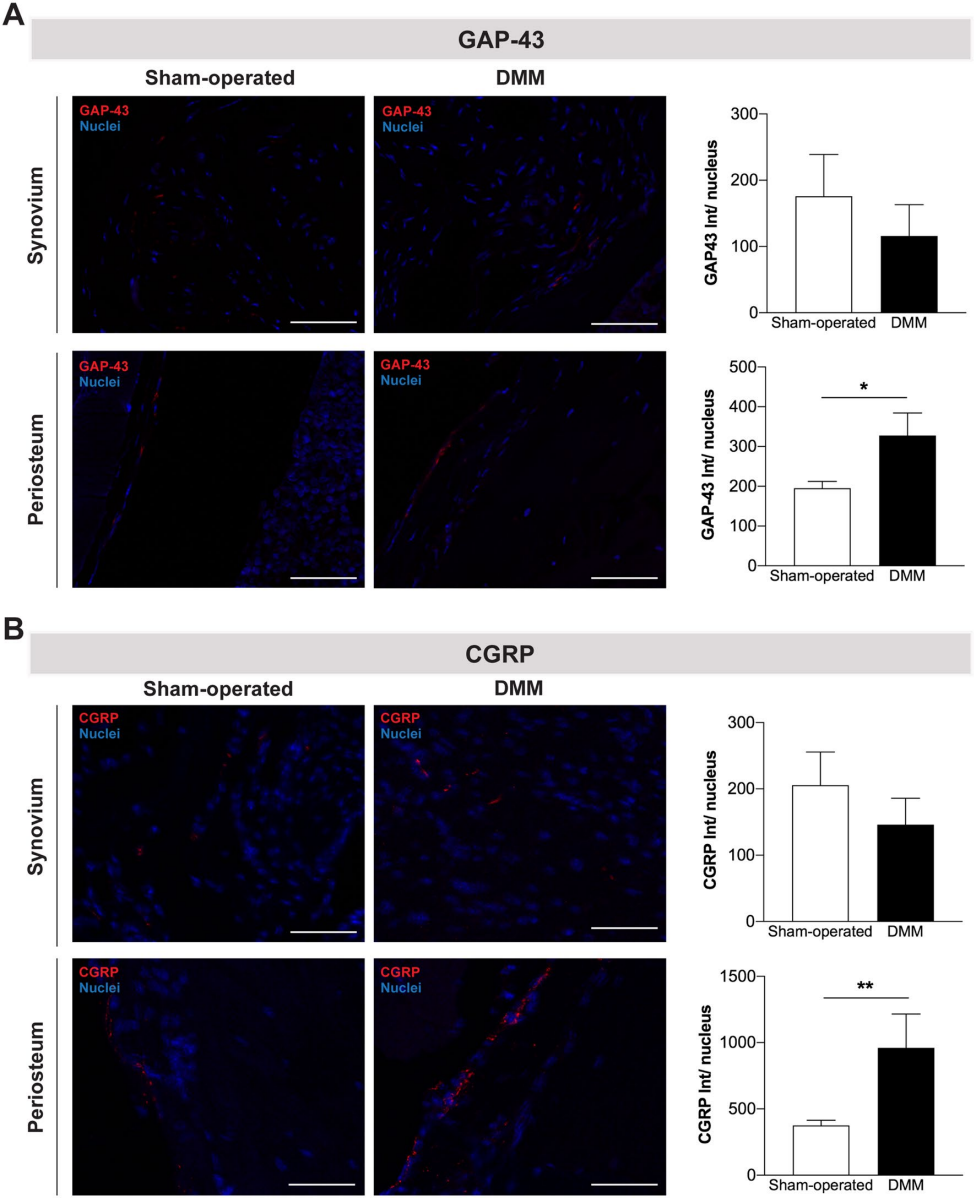
CGRP innervation was increased in the periosteum after the induction of the OA post-traumatic DMM model. The presence of nerve fibers positive for GAP-43 (**A**) and positive for CGRP (**B**) was assessed in the knee joint 12 weeks post-surgery. No alterations in the GAP-43 and CGRP innervation patterns were observed in the synovium of DMM mice, however, increased GAP-43 and CGRP innervation was observed in the periosteum of these mice. Scale bar = 50 μm. Results are presented as mean ± SEM, n = 5 per group. *p < 0.05, **p < 0.01.

Contrary to the lack of innervation in the articular cartilage of healthy joints, sensory and sympathetic nerve fibers have been observed in the articular cartilage of mild and severe human OA stages^15^. It has been hypothesized that the changes in joint innervation might be related to pain severity^16^. Here, we evaluated the presence of nerve fibers positive for growth-associated protein (GAP)-43 (a marker of neurons growth and regeneration) in the knee joint structures. We observed no GAP-43 positive nerve fibers innervating the cartilage (data not shown) and no alterations in the GAP-43 innervation profile of the synovium of the DMM mice (Fig. 3A). Nevertheless, increased GAP-43 innervation was observed in the periosteum of these mice when compared with sham-operated mice (p = 0.0357).

Data from clinical and animal OA studies highlighted a crucial role of sensory innervation in OA pain^16–19^*.* In this study, we investigated changes in the sensory innervation through the evaluation of the CGRP innervation pattern of the joint. We found no CGRP-nerve fibers invading articular cartilage (data not shown) and no alterations in the CGRP innervation profile of the synovium of the DMM mice. However, as for the GAP-43, we observed an increase in the density of CGRP-nerve fibers innervating the periosteum of the DMM mice (p = 0.027) (Fig. 3B).

### Alteration in peripheral nociceptive pathways in the OA post-traumatic DMM model

**Figure 4:**
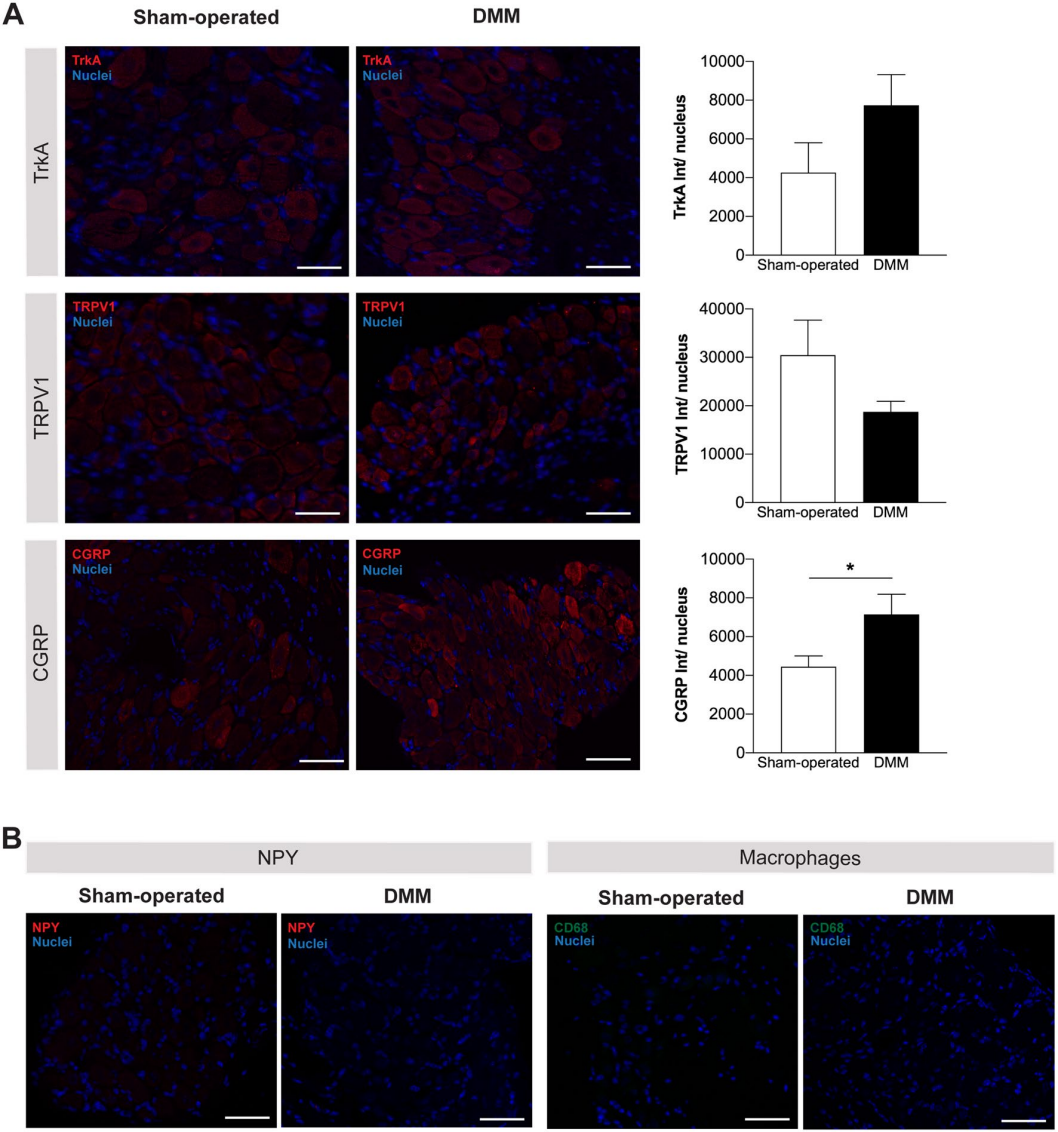
CGRP expression was increased in the DRG after the induction of the OA post-traumatic DMM model. The expression of TrkA, TRPV1, and CGRP (**A**) was assessed in the DRG 12 weeks post-surgery; quantitative analyses of the staining intensity were performed. The expression of NPY in DRG and the infiltration of DRG by macrophages were also assessed at the same time point of disease progression (**B**). Increased expression levels of CGRP in the DRG of DMM mice were observed at 12 weeks after the knee traumatic lesion. No differences were observed in the expression of TrkA and TRPV1 between DMM and sham-operated mice. No expression of NPY was found in the DRG, and no macrophages were infiltrating the DRG 12 weeks after DMM surgery. Results are presented as mean ± SEM, n = 5 per group. *p < 0.05. Scale bar = 50 μm.

In addition to the structural and biochemical changes in the injured joint, alterations in the peripheral and central pain signalling pathways are key components of OA pain^10^. Both pre-clinical and clinical studies have implicated the nerve growth factor (NGF)/receptor Tyrosine Kinase A (TrkA) signalling pathway in the OA pain, and its antagonism was shown to result in OA pain reduction^19–22^. The NGF released by injured tissues activates TrkA in sensory neurons, up-regulating the neuropeptidergic signalling (by CGRP and SP) and transient receptor potential cation channel subfamily V member 1 (TRPV1), pain-related molecules involved in peripheral sensitization^23,24^. Here, we investigated alterations in the TrkA, CGRP, and TRPV1 expression in the DRG (where the cell bodies of sensory neurons are located). Our data showed increased expression of CGRP in the DRG of DMM mice (p = 0.0401) (Fig. 4A). No alterations were observed in the expression of TrkA and TRPV1 (Fig. 4A).

We also assessed the expression levels of neuropeptide Y (NPY), a marker of neuropathic pain that is expressed in DRG only in response to nerve injury^25,26^. As above described the innervation by GAP-43 is up-regulated in the periosteum, which could suggest nerve damage in the DMM mice. However, we observed no detectable expression of NPY in the DRG at 12 weeks post-DMM surgery (Fig. 4B).

In this study, we also investigate the infiltration of DRG by macrophages. This mechanism has been highlighted as playing an important role in the initiation and maintenance of peripheral neuron sensitization in neuropathic and inflammatory pain models^27^. However, here we found no macrophages infiltrating the DRG of DMM mice (Fig. 4B).

### Markers of central sensitization in the OA post-traumatic DMM model

**Figure 5:**
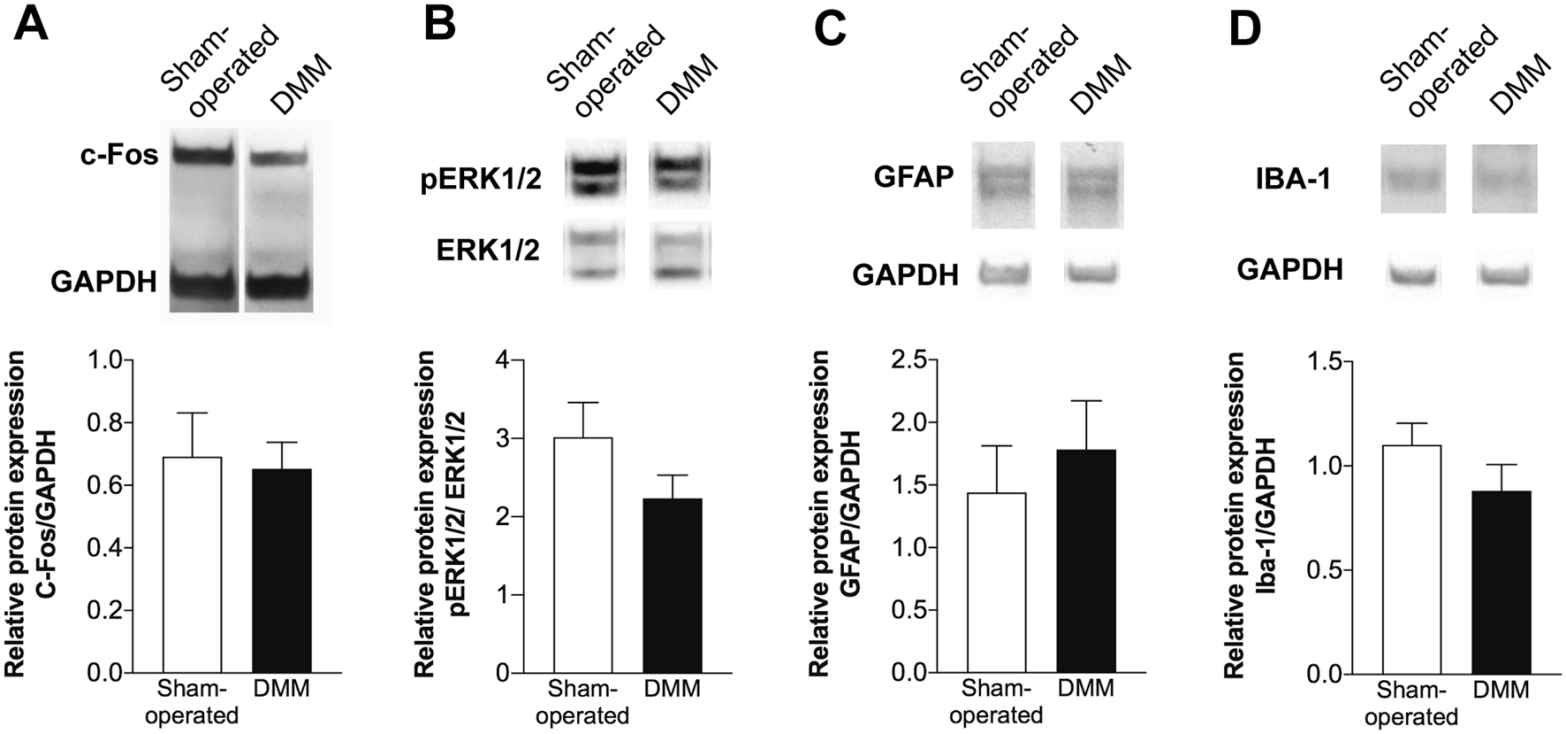
Markers of central sensitization were not altered in the OA post-traumatic DMM model. The protein levels of c-Fos (**A**), p-ERK1/2 (**B**), GFAP (**C**) and IBA-1 (**D**) were quantified in the spinal cord of the DMM mice 12 weeks after surgery by western blot. The p-ERK1/2 expression was normalized to ERK1/2, and the c-Fos, GFAP and IBA-1 expression were normalized to the GAPDH. No differences were observed in the expression levels of both markers between DMM and sham-operated mice. Results are presented as mean ± SEM, n = 5 per group.

It was reported that up to 80% of the OA patients achieve pain relief with peripheral analgesia, supporting a prevalence of peripheral mechanisms driving OA pain^28,29^. However, centrally mediated pain sensitization has also been reported in some OA patients^30^*.* Central sensitization results from the excessive activity of peripheral nociceptive pathways, that leads to increased release of excitatory molecules, including the CGRP, into the spinal cord dorsal horn contributing to the sensitization of the second-order pain transmission neurons^31^. In this study, we investigated whether the observed increase in the CGRP levels in the DRG of DMM mice could have contributed to the establishment of central sensitization. To assess this, the protein levels of c-Fos and phospho-extracellular signal-regulated kinases 1/2 (p-ERK1/2), markers of neuronal activation, and the levels of ionized calcium-binding adapter molecule 1(IBA-1) and glial fibrillary acidic protein (GFAP), known to be upregulated in activated microglia and astrocytes^32,33^, were quantified in the spinal cord. DMM mice presented no alterations in the expression levels of these markers (Fig. 5).

## Discussion

In the present study, we demonstrated that the expression of CGRP is augmented both in the sensory nerve fibers of the periosteum and in the DRG, at 12 weeks after the traumatic injury of the knee joint in the DMM model. This increased CGRP expression might be underlying the peripheral pain sensitization, reflected by an increase in mechanical allodynia. Additionally, at this time point of OA progression, our observations revealed that the infiltration of the synovium by immune cells, and neuropathic and central sensitization mechanisms did not contribute to the displayed pain behaviour.

The marked cartilage damage, the narrowing of the joint space, and the presence of osteophytes and subchondral bone sclerosis support the development of an OA phenotype at 12 weeks post-DMM surgery. However, the inflammation of the synovial membrane, another common feature of OA^34^, seems not to have a critical role on the pathophysiology of this preclinical model at this stage of the disease progression. In fact, we did not observe infiltration of the synovium by immune cells. Using the DMM model, Driscoll et al. also reported no increase in joint inflammation 12 weeks post-surgery^35^. Together, these results corroborate previous studies describing an initial intense inflammatory response to the traumatic joint injury, which is followed by lower inflammation levels in later phases^36^.

Along with the observed morphological and histological alterations, mice developed mechanical allodynia. This supports previous studies reporting the presence of mechanical allodynia in this OA model^9^ and indicates the development of pain sensitization^37^.

Decreased locomotor activity and altered gait pattern are also a common consequence of OA pain. The critical time point for alteration in the locomotor activity in post-traumatic OA models is not clearly defined. Sambamurthy et al. reported altered locomotor activity at week 8, when comparing DMM animals with non-operated controls, but not when comparing with sham-operated animals^38^. On the other hand, Miller et al. described decreased locomotor activity in DMM mice at week 8, but at week 16 this effect was no longer observed^39^. In this study, we observed no differences in the locomotor activity between DMM and the control groups (non-operated and sham-operated mice), indicating that, until the week 12 post-DMM surgery, pain is not affecting the locomotor activity. Changes in gait parameters, when using the CatWalk system, have been reported to emerge between 10 and 12 weeks post-DMM surgery^40,41^. Here, we evaluated putative changes in the gait pattern following the method described by Boettger et al.^42^, and none of the assessed measures were different between DMM and the non-operated and sham-operated controls. However, we observed a decrease in the ITS in the affected paw of DMM mice, suggesting that pain was interfering with the foot placement.

Overall, the OA post-traumatic DMM model is characterized, at 12 weeks progression stage, by a marked knee joint damage that was associated with mechanical allodynia but not associated with alterations in locomotor activity and gait pattern.

The normally aneural articular cartilage of the healthy joints, is described to become innervated by sensory and sympathetic nerve fibers in mild and severe human OA stages^15^. It has been hypothesized that the changes in joint innervation might be related to pain severity^16^. Contrary, a reduction of the sensory innervation in the synovium was reported in human samples, and was shown to be closely related with inflammation^43^. Decrease synovial sensory innervation was also described in inflammatory collagenase-induced OA mouse model^44^. In the present study, 12 weeks after the DMM surgery we observed no sensory nerve fibers in the cartilage and no differences in the sensory innervation of the synovium. The lack of differences in the synovial sensory innervation might be related with the low inflammatory nature of the DMM model. However, we observed alterations in the innervation pattern of the periosteum.

The increased expression of neuronal GAP-43 in the periosteum that we observed in the DMM mice indicates nerve sprouting in this area. We hypothesized that these growing nerve fibers were probably mainly sensory, as we also observed an increase in the number of CGRP-positive nerve fibers in the periosteum. CGRP, a neuropeptide found primarily in the C and Aδ sensory fibers*,* is well known to modulate neuronal sensitization and increase pain. To our knowledge, we are the first reporting sprouting of sensory innervation in the periosteum in a post-traumatic OA model. The periosteal sensory innervation has been highlighted as a critical player in bone pain. The increase in the density of CGRP nerve fibers in periosteum has been reported associated to pain in bone pathological conditions such as non-healed fracture^45^ and bone cancer^46,47^. The increase in the periosteum innervation by CGRP-positive nerve fibers was also previously reported in Complete Freund's adjuvant (CFA)-induced arthritis in mice^48^ and in and the Monoiodoacetate (MIA) rat model^49^. Importantly, the blockade of the sprouting of the periosteal sensory innervation, by the administration of an anti-NGF antibody, is associated with the inhibition of the pain-related behavior in bone cancer^46,47^. Together with our results, these data support the sensory innervation of the periosteum as an important potential source of pain in post-traumatic OA.

The CGRP expressing neurons are responsive to NGF through its action on TrkA^31^. The activation of the NGF/TrkA signalling pathway induces the up-regulation of CGRP and other molecules such as TRPV1, both known to be involved in pain sensitization^23,24^. Importantly, both pre-clinical and clinical studies show that the blockage of NGF signalling produces analgesia in OA pain^19–21^. The up-regulation of CGRP, TRPV1, and TrkA in DRG was previously described in MIA-induced OA model^50,51^. Here, we report an increase in the DRG expression of CGRP, but no alterations in the expression of TrkA and TRPV1. The different observations between MIA-induced and DMM models may be related to the different prevalence of the inflammatory mechanisms between these two models.

In this study, we hypothesized that the increased expression of CGRP in the sensory nervous system is involved in the mechanisms underlying the mechanical allodynia presented by the DMM mice. Our hypothesis is in line with previous data showing that exogenous administration of CGRP to the knee mimics the peripheral sensitization of arthritic joints, which is decreased by CGRP antagonisms^52^. In fact, the targeting of CGRP signalling in arthritic joints has been highlighted, in animal studies, as a putative strategy to treat pain^52,53^.

Further studies are needed to unveil the mechanisms underlying the increase in the CGRP expression and other related mechanisms of peripheral sensitization, from the receptors expressed on the nociceptors membrane, to the activated intracellular pathways (e.g. PKC, PKA, PI3K, the MAP kinases ERK and p38, and JNK) and the downstream effectors of peripheral sensitization (e.g. the phosphorylation of TRP and voltage-gated channels, such as TRPV1, TRPA1 VGSCs, Nav1.8, Nav1.7, and Nav1.9).

In addition to pain sensitization, neuropathic pain mechanisms were also described in some chemically-induced OA models, for instance in the MIA model^54^. Neuropathic pain is defined as the pain caused by lesion or disease of the somatosensory system^55^. Although we had observed an increase in the GAP-43 positive-nerve fibers in the DMM mice, which could suggest nerve damage, we found no DRG expression of NPY, which is a marker of neuropathic pain expressed in DRG only in response to nerve injury^25,26^. Therefore, our results suggest that, at this stage of OA progression post-DMM surgery, the neuropathic mechanisms were not a significant component of OA pain. The low contribution of a neuropathic component is further supported by the observed absence of macrophages invading the DRG. Macrophages are typically found in DRG after nerve injury, as observed in the constriction or transection of the sciatic nerve models^56,57^. In inflammatory joint models, macrophages are also observed invading DRG^58–60^. Here, the absence of macrophages in DRG is in line with our observation of a putative low inflammatory response. Overall, the neuropathic and the inflammatory components did not have a significant role in the pain displayed at 12 weeks post-DMM surgery.

Centrally mediated pain sensitization has been reported in OA patients^30,61^ and animal models^62,63^. But, to our knowledge, this mechanism was not previously studied in the DMM model. Peripheral sensitization leads to the increased release of excitatory molecules by the sensory neurons, including the CGRP, into the spinal dorsal horn, contributing to the sensitization of the second-order pain transmission neurons^31^. In the MIA-induced OA model, pain development is correlated with an increase in the c-Fos and p-ERK1/2 (markers of neuronal sensitization^64,65^) in neurons of the spinal cord dorsal horn^66,67^. Moreover, the intrathecal injection of the ERK1/2 signalling pathway inhibitor PD98059 reduces the pain and the p-ERK1/2 induction in this OA model^66^. In the CIA-induced arthritis model the analgesia achieved by the intrathecal administration of tramadol also show the reduction in the p-ERK1/2 increase^68^. The spinal microglia and astrocytes were shown to play a critical role in development of the pain hypersensitivity, as they can dynamic modulate the activity of spinal neurons^69,70^. This has been demonstrated namely in models of arthritis, such as collagenase-induced^71^ and CFA-induced arthritis^72^. The involvement of microglia and astrocytes is strongly supported by the data showing that their inhibition decreases nociception^71,72^. Here, we observed that the spinal levels of c-Fos and p-ERK1/2, as well as, IBA-1 and GFAP (upregulated in activated microglia and astrocytes, respectively) were not affected. These results indicate no neuronal sensitization or activation of microglia and astrocytes, and therefore, no development of mechanisms of central sensitization.

Our data revealed that there was an increased expression of CGRP in the sensory nervous system 12 weeks after the traumatic knee joint injury, which we hypothesized to be underlying the observed peripheral pain sensitization. Features previously described in other OA models, such as the infiltration of synovial membrane by immune cells, development of neuropathic mechanisms and central pain sensitization were not observed in the OA post-traumatic DMM model. Therefore, our results suggest that the nociceptive mechanisms involved in driving pain in post-traumatic OA are considerably different from those in non-traumatic OA. These findings highlight an important demand to adjust the therapeutic approaches to the nociceptive mechanisms driving pain in post-traumatic OA and uncover the modulation of the CGRP expression as a putative therapeutic approach.

## Methods

### DMM surgery

Mice were subjected to volatile anaesthesia with isoflurane, and under the stereomicroscope, a longitudinal incision medial to the patellar ligament was performed in the right leg and the joint capsule was opened. The meniscotibial ligament, anchoring medial meniscus to the tibial plateau was transected, resulting in the destabilization of the medial meniscus, as previously described^8^. The joint capsule and subcutaneous layer were sutured, and the skin was closed by intradermal suture. The sham-operated mice underwent the same surgical procedure but the meniscotibial ligament was left intact. Analgesia was provided by the administration of 0.08 μg/g body weight of buprenorphine (Bupaq^®^, Richer Pharma AG, Wels, Austria) on the surgery day and the following 3 days. At 12 weeks post-surgery, mice were euthanized with an overdose of anaesthetic and tissues were collected (hind limbs, ipsilateral lumbar L2–L5 dorsal root ganglia (DRG) and spinal cord).

### X-ray

Animals were subjected to volatile anaesthesia with isoflurane and X-ray images were obtained at 12 weeks post-DMM surgery using the X-Ray Owandy system (Croissy-Beaubourg, France).

### Pain-related behaviour, locomotor activity, and gait pattern

#### Mechanical allodynia

The Von Frey test was used to evaluate mechanical allodynia at 4, 6, 8, 10 and 12 weeks post- surgery in sham-operated and DMM mice. Data from a non-control group was also obtained. The mice were placed individually in small cages over a hire mesh platform and allowed to acclimate for 30 min. Whenever the exploratory behaviour was not reduced during this period, the animals were allowed to acclimate for an extra period until the activity was reduced. The filaments were applied perpendicularly to the plantar surface of the ipsilateral hind paw until it bowed and then held for 4 s, following the up-down method^73^. Briefly, a series of Von Frey filaments (Ugo Basile, Varese, Italy) were applied with increasing bending forces (ranging from 0.04 to 1.4 g), beginning with the 0.04 g filament and until paw withdrawal response (positive response) was observed. The withdrawal threshold was recorded to the smallest filament to evoke at least 3 responses out of 5 repeated applications. When a positive withdrawal response was obtained with 0.04 g filament, the paw was retested starting with next descending Von Frey hair until no response occurred.

#### Locomotor activity

Sham-operated and DMM mice were subjected to the open field test at 4, 6, 8, 10 and 12 weeks post-surgery, and spontaneous locomotor activity was analysed. Data from a non-operated group was also obtained. The apparatus consisted of an empty square arena (40 × 40 × 40 cm), illuminated by indirect white lighting (100 lx). Each mouse was placed in the centre area and allowed to walk freely for 10 min. The following behaviours were automatically recorded by the Smart Video Tracking Software (Panlab) version 3.0: (i) total distance travelled (ii) mean velocity (excluding the immobility time) and (iii) immobility time.

#### Gait pattern

Mice hind paws were coloured with ink, and animals were immediately allowed to cross a tunnel (10 cm wide and 60 cm long, that ended in a dark chamber to entice the mice) leaving their paw prints on a white paper. Data was obtained at 4, 6, 8, 10 and 12 weeks post-surgery from the non-operated, sham-operated and DMM mice. The following measures were collected as previously described by Boettger et al.^42^: the distance between a print from the right paw and a consecutive print from the right and left paw (right-right and right-left distance, respectively) and the angle between consecutive paw prints (i.e. rotation of paws). To exclude the mice speed as a confounding factor, the distance between the right (affected) paw print and the consecutive left one was normalized to a complete walking cycle (right-to-right-distance), and this parameter is referred to as “relative step length”^42^. Additionally, footprint analysis was used to study paw posture, and parameters such as toe spread (TS), intermediate toe spread (ITS) and print length (PL) were also collected^74^.

### μ-CT analysis

The right knee joints were scanned at 70 kV, 200 mA with a 0.5 mm aluminum filter and an isotropic resolution of 8 mm, using a Skyscan 1276 System (Brucker, Belgium). The obtained projection images were reconstructed with NRecon software, followed by bone alignment along the sagittal axis using DataViewer software. Morphological analysis was carried out at the medial and lateral tibial condyle (at 50 slices for each side) with CTAnalyser software. Following the recommended guidelines ^75^, the parameters calculated included: mineral density, total volume, and thickness of the subchondral bone. Representative three-dimensional images were generated using CTVol software.

### Histological analysis

After fixed in 4% neutral buffered formalin for 24 h, limbs were decalcified in 10% formic acid for 1 week at 4 °C on a constant shaker, and processed for paraffin embedding. Serial 3 μm thick sections from 5 animals in each group were obtained in the microtome (RM2255, Leica Biosystems) and stained with Safranin O to evaluate cartilage integrity. After deparaffinising, the sections were rehydrated with descending concentrations of alcohol and stained with haematoxylin for 2 min, followed by staining in Fast green for 5 min, washed with 1% acetic acid and counterstained with 1.5% Safranin O for 30 min. Sections were then dehydrated in absolute alcohol, cleared in xylene and mounted under coverslips using DPX mounting medium (VWR, UK). Images were acquired with an Olympus CX31 light microscope equipped with a DP-25 camera (Imaging Software CellB, Olympus, PA, USA). The Osteoarthritis Research Society International (OARSI) scoring system was used to evaluate histological changes of articular cartilage in the medial femoral condyle and tibial plateau^76^. For each knee, the maximum OA score was determined by the lesion with the highest score.

### Immunohistochemistry analyses

#### Knee

For the evaluation of the innervation profile of the knee structures, serial knee sections from 5 animals in each group, obtained as described in “Histological analysis” section, deparaffinised and rehydrated were placed in antigen retrieval for 20 min at 97 °C (10 mM citrate buffer, pH 6.0). After quenching endogenous fluorescence with 0.1% NaBH_4_ and 100 mM NH_4_Cl, sections were incubated with blocking buffer (10% Fetal bovine serum (FBS), 1% Bovine serum albumin (BSA), 0.2% Triton X-100) for 1 h at room temperature (RT), and then incubated overnight at 4 °C with rabbit anti-CGRP (1:6,000, Sigma-Aldrich, USA) or rabbit anti-GAP-43 (1:1,000, Sigma-Aldrich, USA) diluted in blocking buffer at 4 °C.

For the evaluation of knee joint tissue infiltration by immune cells such as macrophages (CD68), B cells (CD20) and T cells (CD3), after antigen retrieval (20 min at 97 °C in 10 mM citrate buffer at pH 6.0), immunohistochemistry was carried out using the M.O.M Basic Kit (Vector, Peterborough, UK) following the manufacturer's instructions. Knee sections from 5 animals in each group, were incubated overnight at 4 °C with the mouse anti-CD68 (clone 514H12, 1:100, Novocastra, UK), mouse anti-CD20 (clone L26, 1:100, Cell Marque, USA), and mouse anti-CD3 (clone PS1, 1:100, Biocare Medical, USA) diluted in M.O.M buffer. To investigate the expression of the macrophages maker F4/80, knee section from 5 animals in each group were incubated for 10 min. at 37 °C in proteinase k for antigen retrieval, and after blocking in 10% FBS, 1% BSA and 0.2% Triton X-100 for 1 h at RT, were incubated overnight at 4 °C with the rat anti-F4/80 (clone BM8, 1:50; BioLegend, USA).

In all experiments, for signal detection, tissue sections were incubated for 1 h at RT with anti-rabbit Alexa Fluor 568 antibody, or anti-mouse Alexa Fluor 488 antibody or anti-rat Alexa Fluor 488 antibody at 1:1,000 (Life Technologies, USA). Nuclei were stained with DAPI and tissue sections mounted with Fluoromount Aqueous Mounting Medium (Sigma-Aldrich, USA). Images were acquired on the confocal Leica TCS SP5 microscope (Leica Microsystems, Germany).

#### Dorsal root ganglia

The expression of peripheral sensitization-related molecules such as CGRP, TRPV1, NGF, TrkA, and of the NPY was evaluated in DRG sections, from 5 animals in each group, by immunohistochemistry analysis. After deparaffinization, rehydration, antigen retrieval and quenching of endogenous fluorescence, histological sections were blocked at RT, and then incubated with rabbit anti-CGRP (1:6,000, Sigma-Aldrich, USA), or rabbit anti-TRPV1 (1:200, Antibodies-online.com, Aachen, Germany), or rabbit anti-TrkA (1:100, Abcam, UK) or rabbit anti-NPY (1:1,000, Sigma-Aldrich, Germany) overnight at 4 °C. For signal detection, tissue sections were incubated for 1 h at RT with anti-rabbit Alexa Fluor 568 antibody (1:1,000, Life Technologies, USA), incubated with DAPI for the nuclei staining and then mounted with Fluoromount Aqueous Mounting Medium (Sigma-Aldrich, USA). Images were acquired on the confocal Leica TCS SP5 microscope (Leica Microsystems, Germany). The staining intensity was quantified using NIH ImageJ software.

### Western blot analyses

Spinal cord samples were collected and frozen in 2-methylbutane cooled over dry ice and stored at − 80 °C until processing. The protein levels of c-Fos, p-ERK1/2, GFAP and IBA-1 were measured by western blot in the spinal cord from 5 animals in each group. The tissue samples were homogenized in lysis buffer (50 mM Tris/HCl, 150 mM NaCl, 2 mM EDTA, 1% Triton-100, 0.5% NP-40 Alternative) containing Protease Inhibitor Cocktail (1:100, Thermo Fisher Scientific) and Phosphatase Inhibitor Cocktail (1:100, Sigma-Aldrich, USA). The homogenate was incubated on ice during 1 h and was then centrifuged at 16,000×*g* for 10 min at 4 °C. Twenty-five microgram of protein supernatant were separated by acrylamide gel electrophoresis (Run Bolt Mini Gels; Bolt 8% Bis–Tris Plus, Invitrogen), at constant voltage 100 mV for 1 h and then electrotransferred using Invitrogen iBlot 2 Transfer Stacks (iBlot 2 NC Mini Stacks, Invitrogen) at 25 V for 7 min. Non-specific protein binding was prevented by membranes blocking for 1 h with 5% of BSA in TBS-T. Membranes were incubated overnight at 4 °C with mouse anti-c-Fos (1:500, Santa Cruz Biotechnology, USA), rabbit anti-pERK1/2 (1:1,000, Cell Signaling, MA, USA), mouse anti-ERK1/2 (1:1,000, BD Biosciences, CA, USA), rabbit anti-GFAP (1:10 000, Abcam, UK), rabbit anti-IBA1 (1:1,000, Wako, USA) and mouse anti-GAPDH (1:20 000, HyTest, Turku, Finland). After washing, membranes were incubated for 1 h at RT with horseradish peroxidase (HRP)-conjugated goat anti-rabbit IgG and goat anti-mouse IgG antibodies (both at 1:10,000, Santa Cruz Biotechnology, Texas, USA). Immunoreactive proteins were revealed using the enhanced chemiluminescence method (Amersham, ECL Prime Western Blotting Detection Reagent, GE Healthcare, UK) as recommended by manufactured instructions. The bands quantification was performed in Image J software version 2.0. Protein expression is presented as the ratio between the protein of interest and reference protein band densities (GAPDH or ERK1/2). Complete and unprocessed blots can be found in Supplementary Fig. S2.

### Statistic analyses

Data from pain behaviour tests were analysed by a 2-way Mixed Anova analysis followed by Tukey’s multiple comparisons test and data from μ-CT were analysed by 2-way ANOVA followed by Sidak’s multiple comparisons test. All other data were assessed for normal distribution and non-parametric Mann–Whitney analyses were performed whenever normal distribution was not followed. Differences were considered at the significant level of p < 0.05. All data are expressed as mean ± SEM. Statistical analyses were performed using the software Prism 6 (GraphPad software, San Diego, CA, USA).

### Animals

All animal procedures were approved by the i3S animal ethics committee and by the Portuguese Agency for Animal Welfare (Direção-Geral de Alimentação e Veterinária), in compliance with the European Community Council Directive of September 22, 2010 (2010/63/UE). Experiments were conducted by FELASA C and B graded researchers and all efforts were made to minimize the number of animals used and their suffering.

Three-months old C57BL/6 male mice were provided by the i3S Animal Facilities. Mice were kept under controlled conditions (20–22 °C, 60% humidity and 12:12 h light/dark cycle). Water and appropriate food were supplied ad libitum. Animals were randomized into the experimental groups (n = 5).

## Supplementary information

Supplementary Information.

## Abbreviations

OA: Osteoarthritis
BSA: Bovine serum albumin
CFA: Complete Freund's adjuvant
CGRP: Calcitonin gene-related peptide
DMM: Destabilization of medial meniscus
DRG: Dorsal root ganglia
FBS: Fetal bovine serum
GAP-43: Growth-associated protein-43
HRP: Horseradish peroxidase
ITS: Intermediate toe spread
MIA: Monoiodoacetate model
NGF: Nerve growth factor
NPY: Neuropeptide Y
OARSI: Osteoarthritis Research Society International
PL: Print length
RT: Room temperature
TrkA: Tyrosine kinase A
TRPV1: Transient receptor potential cation channel subfamily V member 1
TS: Toe spread
