# Supplementary material for: Nociceptive mechanisms driving pain in a post-traumatic osteoarthritis mouse model
Source: Sci Rep. 2020 Sep 17;10:15271. doi: 10.1038/s41598-020-72227-9 (PMC7499425; doi:10.1038/s41598-020-72227-9)
Supplement: Supplementary file 1 — Supplementary Information. [file 41598_2020_72227_MOESM1_ESM.docx]

**Title:** Nociceptive mechanisms driving pain in a post-traumatic osteoarthritis mouse model

C.J. Alves, M. Couto, D.M. Sousa, A. Magalhães, E. Neto, L. Leitão, F. Conceição, A.C. Monteiro, M. Ribeiro-da-Silva, M. Lamghari


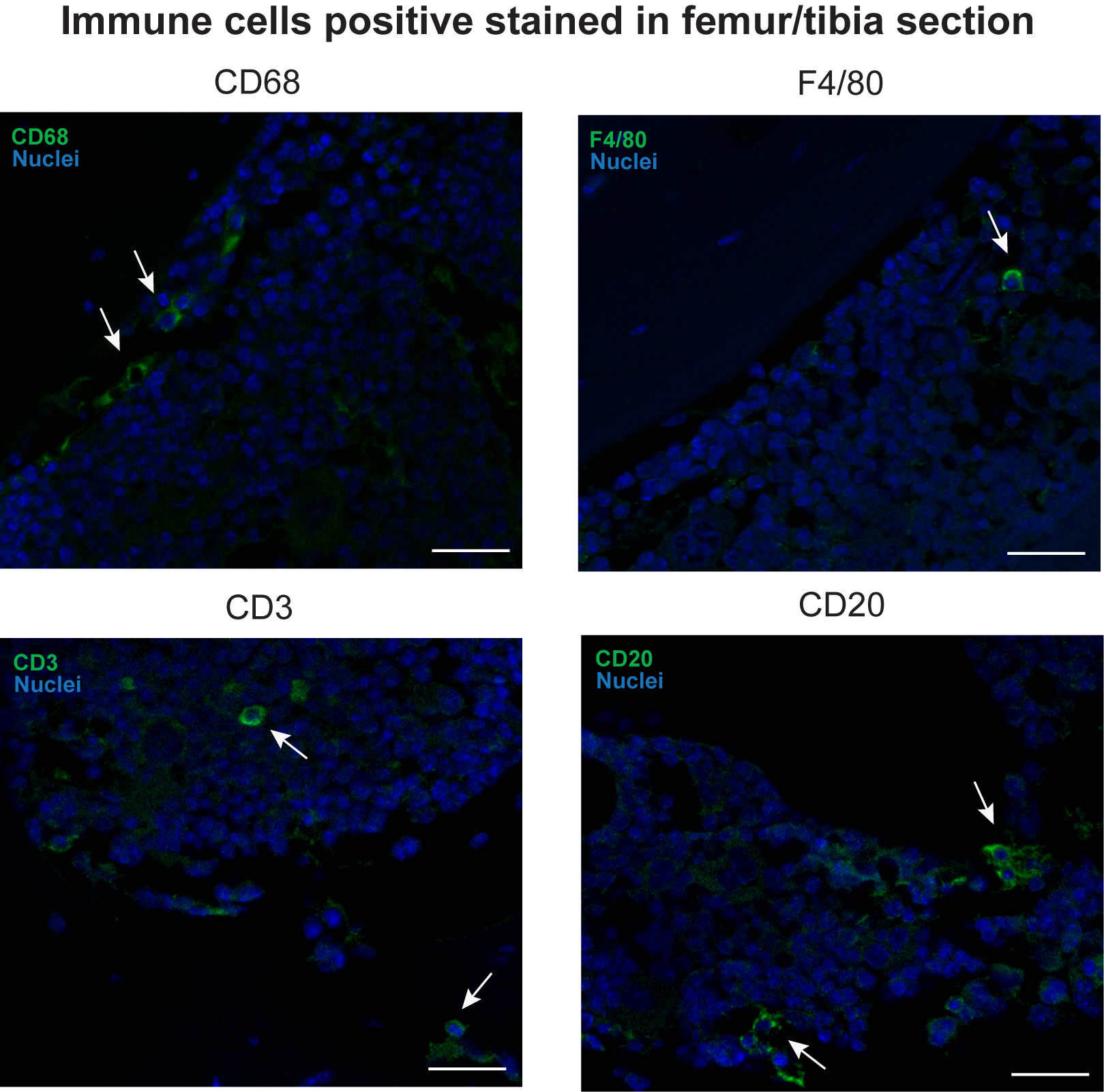


**Figure S1- Positive staining in femur/tibia sections obtained with immunohistochemistry techniques used for the detection of macrophages (CD68 and F4/80), T cells (CD3) and B cells (CD20).** White arrows indicate positive stained cells. Scale bar= 50 μm.


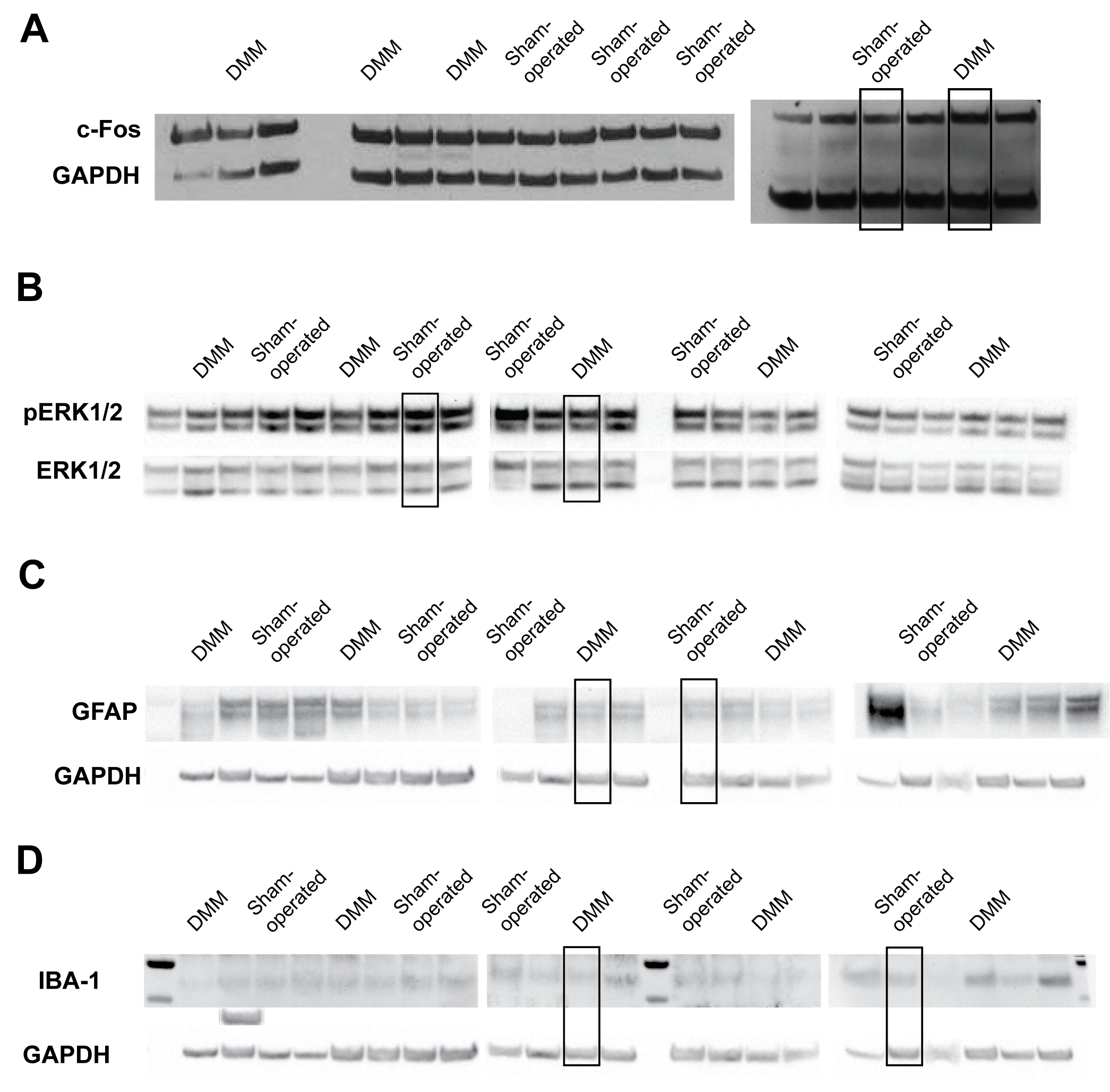


**Figure S2-** Original and unprocessed blots of c-Fos (A), pERK1/2 (B), GFAP (C) and IBA-1 (D) protein expression analyses. Dark rectangles highlight the selected representative bands.
